# RadCube MAGIC observations of complex field aligned currents associated with a supersubstorm during the May 2024 geomagnetic storm

**DOI:** 10.1038/s41598-026-63206-7

**Published:** 2026-07-28

**Authors:** Jonathan P. Eastwood, Martin O. Archer, Cara L. Waters, Harry C. Lewis, Adrian T. LaMoury, Sofia Burne, Kaja Glosli, Richard Baughen, Tim Oddy, Patrick Brown

**Affiliations:** 1 Department of Physics, Imperial College London, London, SW7 2AZ UK; 2 Present Address: School of Physical and Chemical Sciences, Queen Mary University of London, London, EN1 4NS UK

**Keywords:** Space weather, Geomagnetic storm, Supersubstorm, Field aligned current, Magnetometer, CubeSat, Astronomy and planetary science, Climate sciences, Natural hazards, Physics, Planetary science, Solid Earth sciences, Space physics

## Abstract

Geomagnetic storms are a key driver of space weather impacts. The enhancement of ionospheric currents during these events can produce geomagnetically induced currents (GICs) that may interfere with the operation of ground infrastructure such as power grids, pipelines, and railway networks. It is therefore critical to understand how, why, and when very intense GICs may occur during an extended geomagnetic storm interval. Supersubstorms are extremely intense substorms which may occur during the main or recovery phases of geomagnetic storms. These rare events bear special investigation because they may have a significant impact on GIC formation during extreme geomagnetic storms. Multiple supersubstorms were observed during the May 2024 geomagnetic storm, and here we report new observations of supersubstorm field aligned currents (FACs), observed in situ by the MAGnetometer from Imperial College (MAGIC) instrument on the RadCube technology demonstration CubeSat. These measurements provide new insight into the detailed structure of supersubstorm FACs, complementing global FAC maps, and reveal an extended spatial region containing magnetic fluctuations across a broad range of spatial/temporal scales, including intense localised current spikes. As well as revealing new details about supersubstorm-related FACs, these observations illustrate the need for dense constellations of low-Earth-orbit satellite-based magnetometers for space weather monitoring.

## Introduction

Space weather describes the way in which natural solar and space physics phenomena may cause significant socio-economic impact^1,2^. Of particular importance are rare but very severe space weather events, since these pose the most risk to technological systems and form the basis of so-called ‘reasonable worst case scenarios’^3^. Such approaches often consider the impact of a 1-in-100 year (Carrington) event^4^, corresponding to an extreme geomagnetic storm, during which time intense geomagnetically induced currents (GICs) can be produced^5,6^. GICs may interfere with the correct operation of power grids, pipelines, and rail networks^7^. They are considered an important risk pathway because in the case of power grids, they have the documented potential to cause power outages and associated economic impacts^8–10^.

To aid better preparation for, and mitigation of, the effects of GICs, it is important to understand their physical drivers. Field Aligned Currents (FACs), which are generated and controlled by the physics of magnetosphere-ionosphere coupling, are of particular significance^11–13^. This is because FACs are the source of ionospheric electric fields which lead to Pedersen and Hall currents, the latter of which are the auroral electrojets which drive the formation of GICs^5,14^. For improving resilience to space weather, it is critical to identify the mechanisms by which the strongest GICs are generated, and in this context, attention has focussed recently on the role of so-called supersubstorms^15–19^.

Supersubstorms were first identified as particularly intense substorms, where a substorm is considered an “episodic enhancement of nightside auroral activity typically reoccurring every few hours”^13^. Geomagnetic activity is typically characterised using geomagnetic indices, and supersubstorms were defined as isolated and distinct intervals in the SuperMAG SML geomagnetic index^20,21^ where SML ≤ −2500 nT. Supersubstorms are relatively rare, with for example 37 being observed during 1996–2009 (solar cycle 23)^15^. However, they deserve special attention as a study of the Mäntsälä Finland Pipeline has shown that intense GICs were in fact closely associated with supersubstorms^17^.

Further examining the association of supersubstorms with superstorms (characterised by the Dst geomagnetic index, and defined where the minimum Dst ≤ −250 nT), it was found that 82% of superstorms contained supersubstorms, although supersubstorms are not restricted to superstorm intervals. More specifically, in the overall interval of 1981–2012, 49% of supersubstorms were associated with superstorms^16^. However, this study also reported that the occurrence frequency and strength of the supersubstorms did not correlate with the strength of the associated geomagnetic storm. Consequently, the relationship between superstorms and supersubstorms is complex, and despite progress in studying the supersubstorm phenomenon, key questions remain about how they cause GICs, with little known about the structure of FACs during these events.

In this context, the space weather events observed during May 2024 have the potential to provide significant insight^22–26^. During a prolonged period of intense solar wind driving, the resulting geomagnetic storm was the strongest in more than 20 years^27^, and arguably the best-observed storm to date given the availability of a wide range of space- and ground-based data^25^. For example, it has been possible to document space weather impacts on a variety of infrastructure and services including in Canada^28^, USA^29^, Sweden^30^, UK^31^, Ireland^32^, China^33^ and New Zealand^34^.

Within the 10–12 May 2024 geomagnetic storm interval, six supersubstorms have been identified and studied^35^. Here we present a case study of one of these supersubstorms, associated with the arrival of a solar wind pressure pulse, and focussing on new observations of FACs in low Earth orbit made by the RadCube technology demonstration CubeSat^36^. These data provide new information about the structure of supersubstorm field aligned currents, and reveal a complex extended region of magnetic field fluctuations, in addition to localised intense current spikes.

## Results

**Fig. 1:**
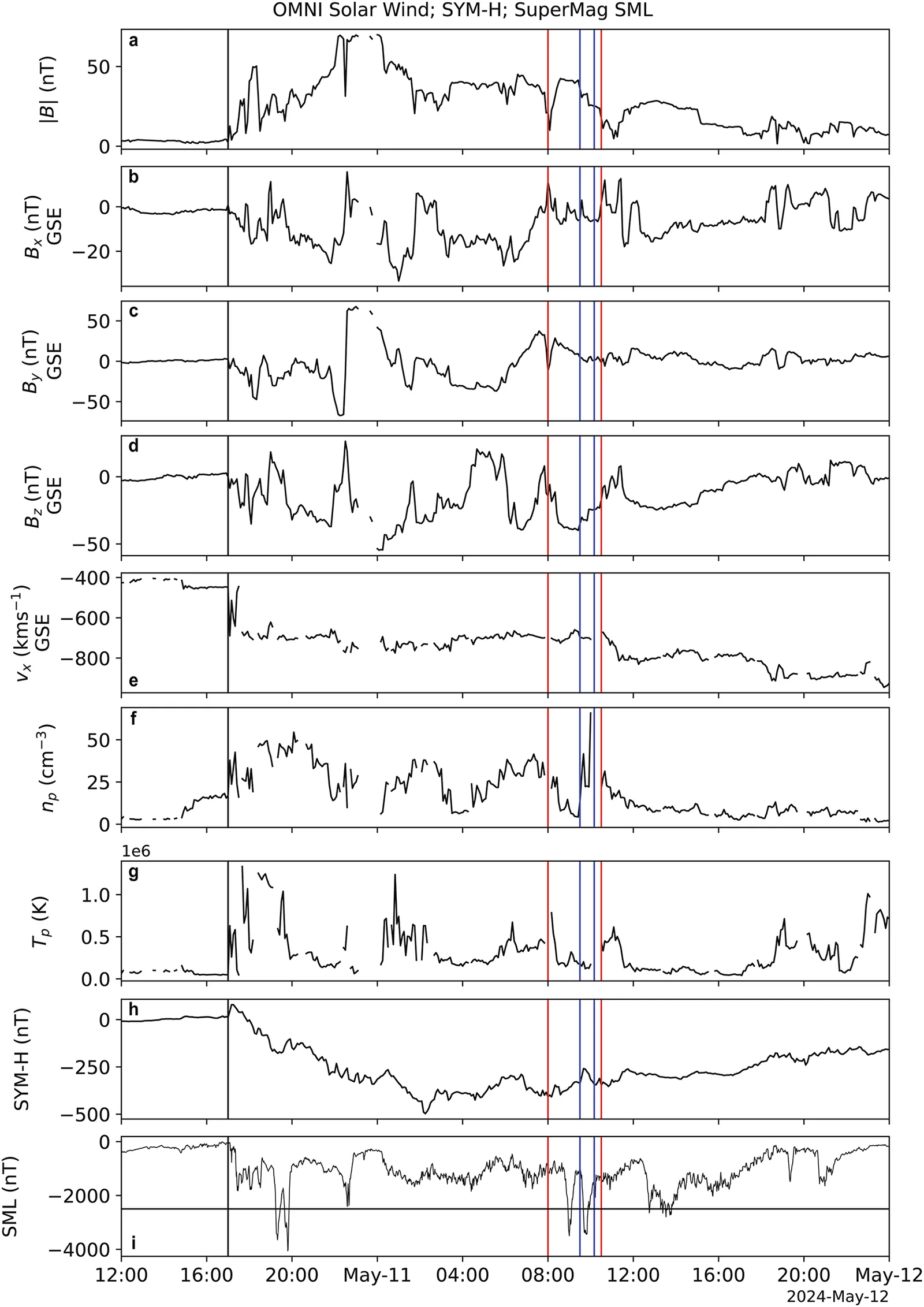
(**a**-**d**) OMNI solar wind magnetic field strength and components in GSE coordinates. (**e**-**g**) OMNI solar wind v_x, GSE_ proton speed, proton density and proton temperature. (**h**) SYM-H geomagnetic index. (**i**) SuperMag SML geomagnetic index. The horizontal black line in panel (**i**) marks the threshold below which a supersubstorm is defined to have occurred. Blue vertical lines mark the supersubstorm that is analysed here, and red vertical lines mark the interval of upstream solar wind shown in Fig. 2(a-h).

An overview of the solar wind conditions and relevant geomagnetic indices is shown in Fig. 1, covering the period 10 May 2024 12:00 UT – 12 May 2024 00:00 UT. Figure 1a-g shows solar wind and interplanetary magnetic field measurements from the OMNI dataset. The onset of the geomagnetic storm is marked by the arrival of an interplanetary shock at 17:00 UT on 10 May 2024 (black vertical line). The subsequent interplanetary magnetic field was highly structured with intervals of southward IMF, corresponding to multiple interacting solar wind drivers evidenced by enhancements in the solar wind proton density and temperature^25^. An unusual feature of this event is that the solar wind speed continued to increase through the interval even as the solar wind magnetic proton density and interplanetary magnetic field strength returned towards the conditions observed at the start of the interval shown in Fig. 1.

The response of the magnetosphere and the evolution of geomagnetic storm conditions is characterised by the SYM-H geomagnetic index shown in Fig. 1h. The arrival of the interplanetary shock causes a sudden impulse which is then followed by a main phase with a minimum value of −518 nT at 02:14 UT on 11 May 2024. This is followed by an extended recovery phase lasting beyond the data shown in Fig. 1. As the geomagnetic storm intensified, two other local minima in SYM-H were observed during the main phase at 19:21 UT and 23:12 UT 10 May 2024 which correspond to structure in the driving solar wind^25^.

Figure 1i shows the SuperMag^21^ SML index^20^, which as discussed above is relevant for monitoring auroral activity. Using the previously mentioned definition of a supersubstorm as SML ≤ −2500 nT (shown as a horizontal black line in Fig. 1i), 6 supersubstorms have been identified during this interval^35^. Their peak intensities were recorded at 19:18 UT and 19:48 UT on 10 May 2024, and at 09:00 UT, 09:47 UT, 12:45 UT, and 13:32 UT on 11 May 2024. We note that the final supersubstorm exhibited a more complex structuring in SML with several intensifications, but it was classed as a single event^35^. Here we focus on the interval 09:30 UT – 10:10 UT 11 May 2024, which is during the recovery phase of the geomagnetic storm and marked by blue vertical lines in Fig. 1. This interval contains a supersubstorm with onset at 09:31 UT, and a peak SML intensity of −3432 nT at 09:47 UT^35^, aspects of which have been reported and studied in Refs^37^ and^38^.

**Fig. 2:**
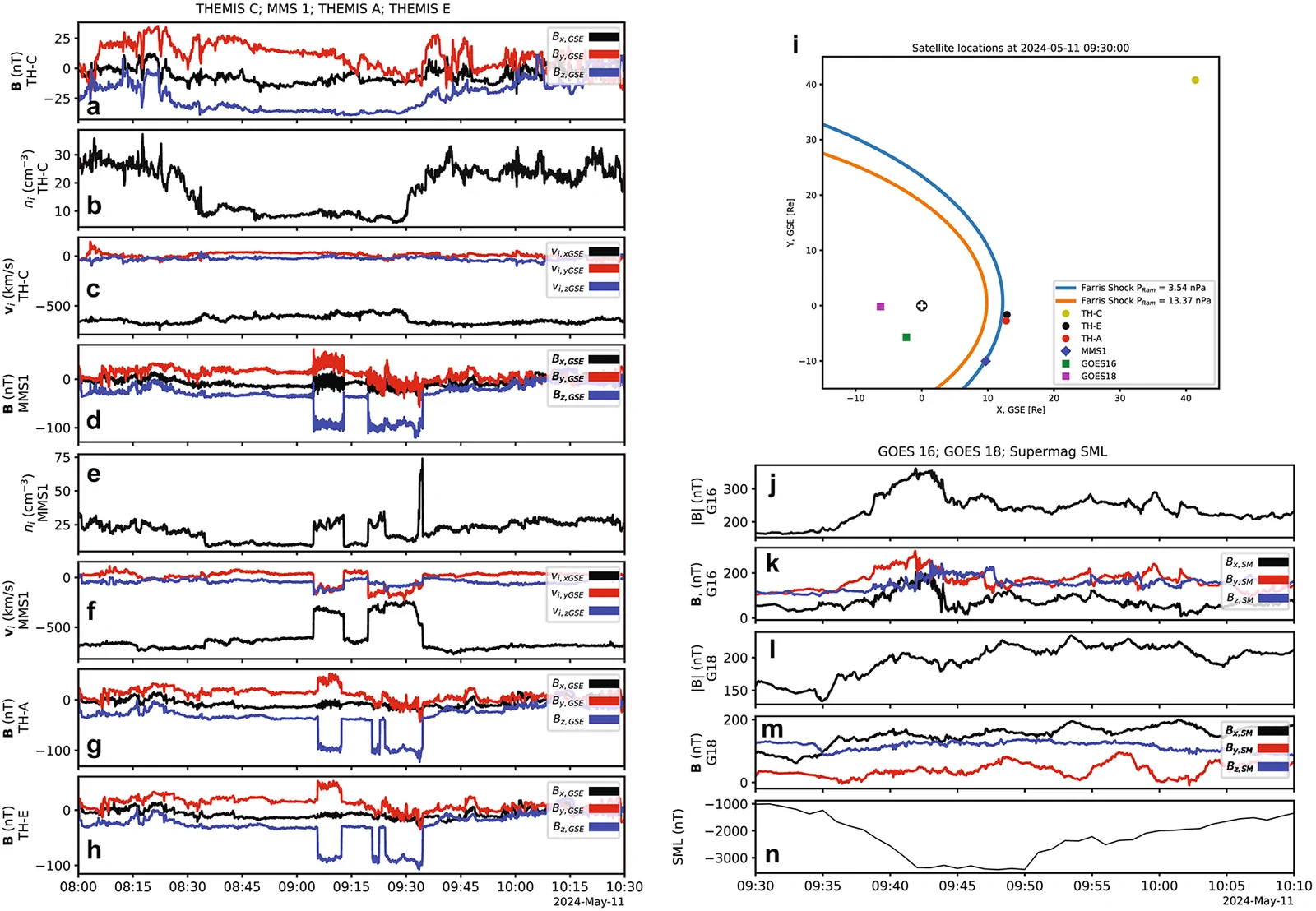
(**a**-**c**) THEMIS-C measurements of magnetic field, ion number density, and ion velocity. (**d**-**f**) MMS1 measurements of magnetic field, ion number density and ion velocity. (**g**) THEMIS-A measurements of magnetic field. (**h**) THEMIS-E measurements of magnetic field. (**i**) Locations of satellites at 09:30:00 UT shown in the X-Y GSE plane and modelled location of the bow shock for solar wind ram pressures observed by THEMIS-C before and after the pressure pulse. (**j**-**k**) GOES 16 magnetic field strength and components. (**l**-**m**) GOES 18 magnetic field strength and components. (**n**) SuperMag SML geomagnetic index.

Figure 2 spans the interval 08:00 UT – 10:30 UT 11 May 2024, marked by red vertical lines in Fig. 1, and provides a more detailed overview of the solar wind context for this supersubstorm. We present data from THEMIS, MMS, and GOES, with the satellite locations projected in the X-Y GSE plane shown in Fig. 2i. Figure 2a-c shows observations from THEMIS-C at [41.5, 40.8, 5.0] Re GSE, in orbit around the Moon and upstream of the dusk flank. An increase in solar wind density and speed was observed by THEMIS-C starting just before 09:30 UT, corresponding to an increase in solar wind ram pressure from 3.5 to 13.4 nPa.

The four MMS spacecraft were located near the bow shock on the dawn flank at [9.7–10.0, −2.2] Re GSE (Fig. 2i). Figure 2d-f shows observations from MMS1, which at 09:30 UT was located in the magnetosheath based on the enhanced magnetic field strength and density, and reduced solar wind speed. Shortly after 09:30 UT, MMS1 observed a large increase in density, as well as an increase in speed. This corresponds to the pressure pulse observed by THEMIS-C, but observed downstream of the bow shock, and is consistent with the expected ballistic propagation time from THEMIS-C to MMS using the observed solar wind speed. Within a few minutes, MMS1 crossed the bow shock back into the solar wind, likely a consequence of the bow shock and magnetosphere responding to the pressure pulse compressing the system^37^. This is confirmed by magnetic field observations from THEMIS-A and THEMIS-E (Fig. 2g-h), both located close to the nose of the bow shock at [12.7, −2.8, −1.6] Re GSE and [12.9, −1.7, −2.2] Re GSE respectively. All three satellites cross from the magnetosheath into the solar wind at similar times, indicating a large-scale change in the position of the bow shock. This is confirmed by the change in the predicted location of the bow shock using the Farris model^39^, for the solar wind ram pressures measured by THEMIS-C before and after the pressure pulse and shown in Fig. 2i.

The magnetospheric response to the pressure pulse was captured by the GOES 16 and GOES 18 satellites and was first analysed by Ref^38^. Magnetic field observations for the interval 09:30 UT – 10:10 UT are shown in Fig. 2j-m together with the SuperMAG SML index in Fig. 2n. GOES 16 was located in the pre-dawn sector and during the main phase of the supersubstorm observed an increase in the magnetic field strength. This is thought to be due to global compression of the magnetosphere by the solar wind pressure pulse^38^. GOES-18, near midnight, observed a decrease in B_z_ and increase in B_x_ at 09:35 UT consistent with magnetotail thinning followed by dipolarization of the magnetic field and increase in magnetic field strength^38^. This occurred at the start of the main phase of the supersubstorm.

Further analysis of this supersubstorm by Ref^37^ suggests that it was accompanied by a rapid intensification of the auroral electrojet that occurred over a wide range of local time and latitude. This led to the conclusion that it was caused by an enhancement of general magnetospheric convection, although the data from GOES-18 may suggest evidence that near-Earth reconnection also could have a contributing role^38^.

**Fig. 3:**
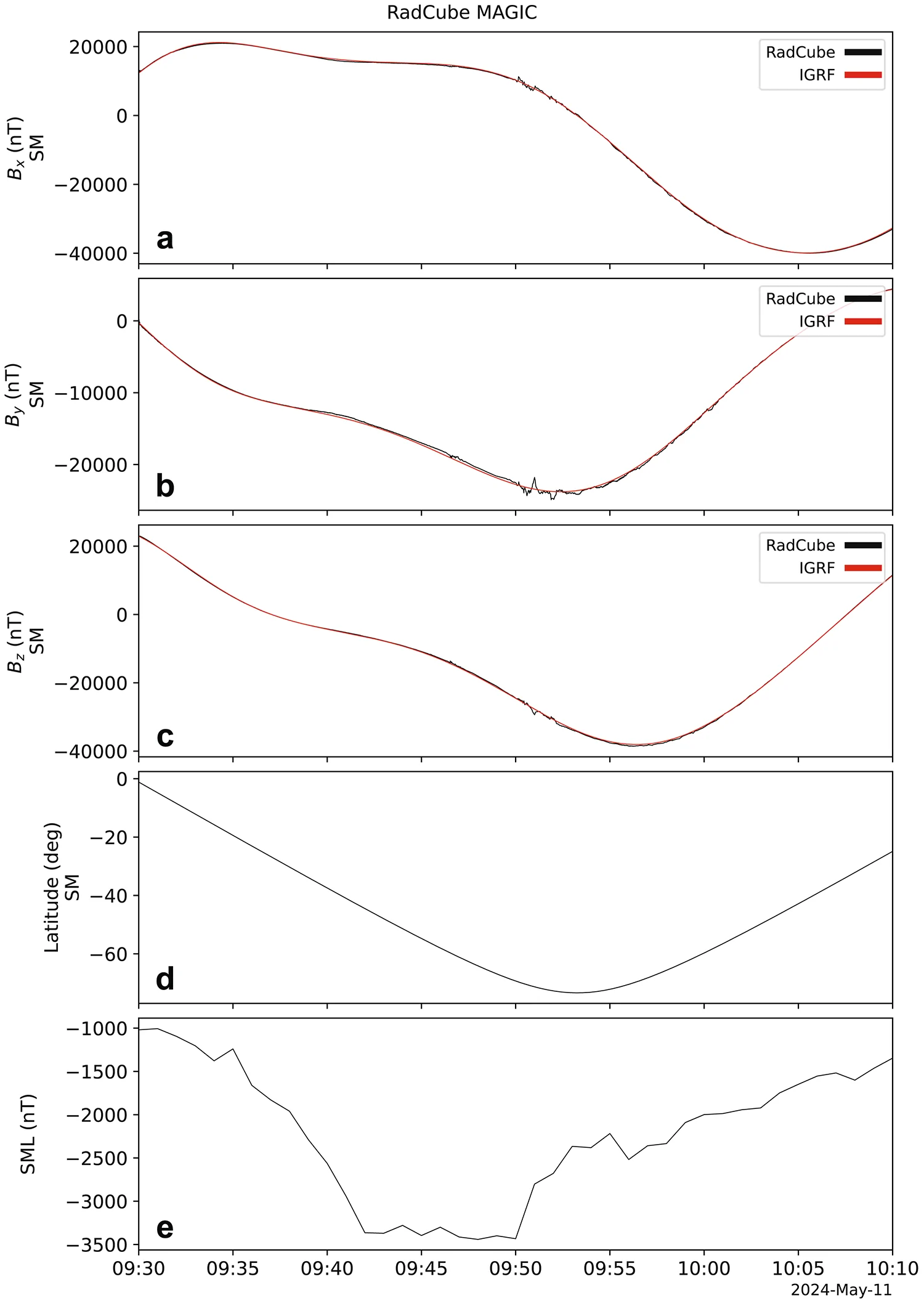
(**a**-**c**) RadCube observations (black) and IGRF model (red) of the B_x_, B_y_, and B_z_ magnetic field components in the SM coordinate system. (**d**) SM latitude of RadCube. (**e**) SuperMag SML geomagnetic index.

Figure 3 shows magnetic field observations from the MAGIC instrument on the RadCube CubeSat, obtained between 09:30 UT – 10:10 UT on 11 May 2024. Figure 3a-c shows the three components of the magnetic field measured by RadCube (black) in the Solar-Magnetic (SM) coordinate system together with the predicted magnetic field from the IGRF-13 model field (red). Even on this scale, structured deviations between the observations and the model field are visible. Figure 3d shows that during this period RadCube was passing over the south pole, and in fact was moving from the dayside to the nightside in its orbit. Figure 3e shows the SuperMAG SML index for context. It should be noted that although SML is derived from northern hemisphere magnetometer stations, the timing of enhanced auroral electrojet activity is expected to be broadly consistent between hemispheres, and so SML is used to identify when in the phase of the supersubstorm the RadCube observations were made. During this interval RadCube started near the equator on the dayside (see Fig. 6) and reached its highest southerly latitudes at the start of the supersubstorm recovery phase. The largest deviations between the observed magnetic field and the model field occurred prior to this at just after 09:50 UT.

**Fig. 4:**
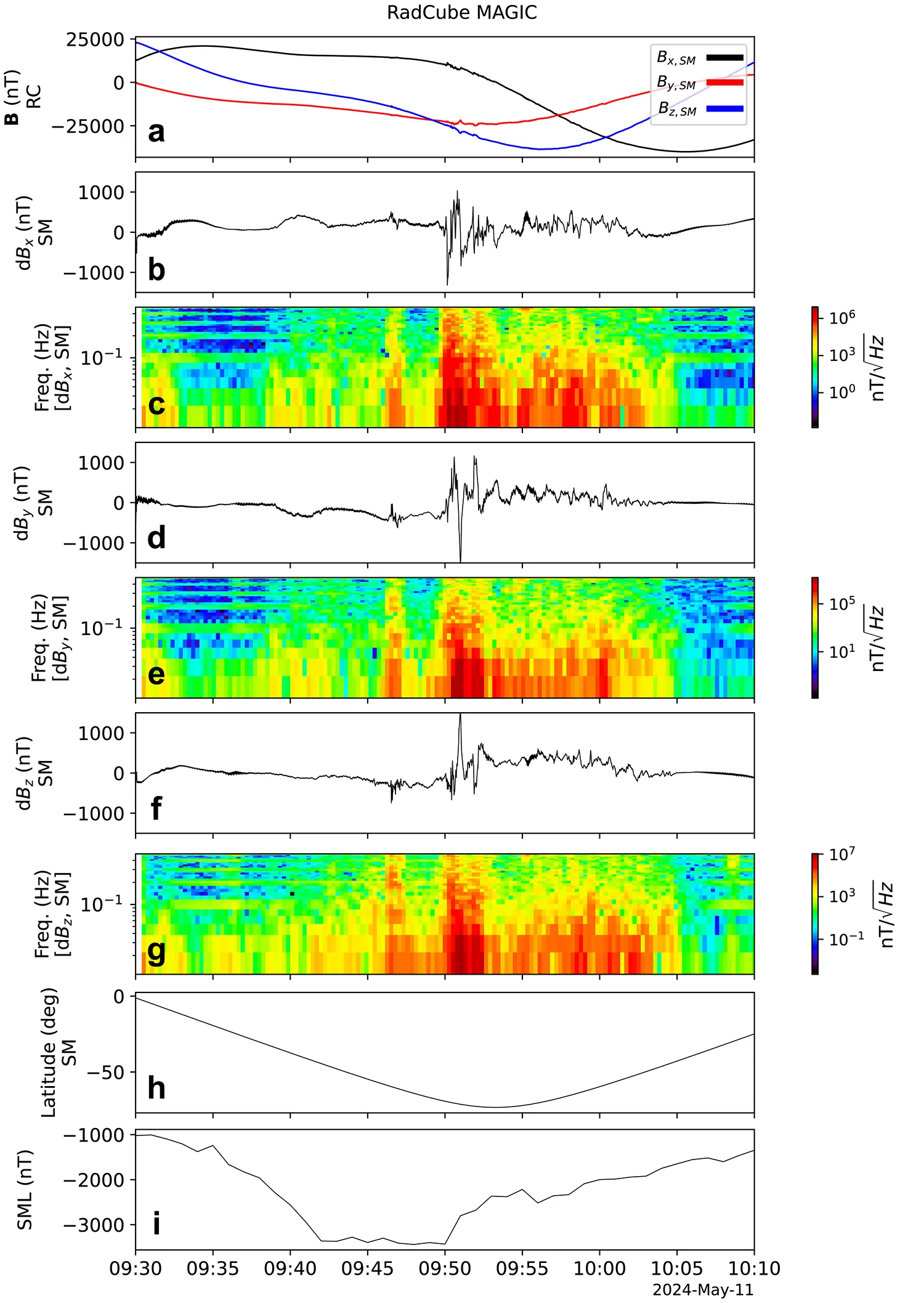
(**a**) RadCube magnetic field measurements in SM coordinate system. (**b**,**d**,** f**) Difference between the RadCube magnetic field measurements and the IGRF-13 model in the x, y, and z SM components. (**c**,**e**,**g**) Power spectral density of the time series shown panels b, d, and f, respectively. (**h**) SM latitude of RadCube. (**i**) SuperMAG SML index.

To explore this further, Fig. 4 shows the differences between the observed magnetic field and the model field in more detail. Figure 4a shows the observed magnetic field in SM coordinates. Figure 4b shows dB_x_ = B_x, RC_ – B_x, IGRF_ where B_x, RC_ is the x-component of the magnetic field measured by RadCube and B_x, IGRF_ is the x-component of the IGRF magnetic field model, both in the SM coordinate system. Figure 4c shows the spectrogram of the data presented in Fig. 4b. Figure 4d-e and f-g show similar data for dB_y_ and dB_z_. For context, Fig. 4h shows the latitude of RadCube and Fig. 4i shows the SuperMAG SML index. Initially the IGRF model well describes the RadCube observations, but during the interval 09:46:20 UT – 09:47:30 UT an initial period of significant deviations in the magnetic field are observed. At 09:50 UT, a series of more substantial magnetic field spikes are observed, with deviations exceeding 1000 nT, lasting until 09:52 UT. Following this, a longer interval of lower amplitude magnetic field fluctuations is observed, lasting until 10:05 UT. The spectrogram of the magnetic field data reveals that this longer interval is associated with power across a broad frequency range in all three components of the magnetic field.

**Fig. 5:**
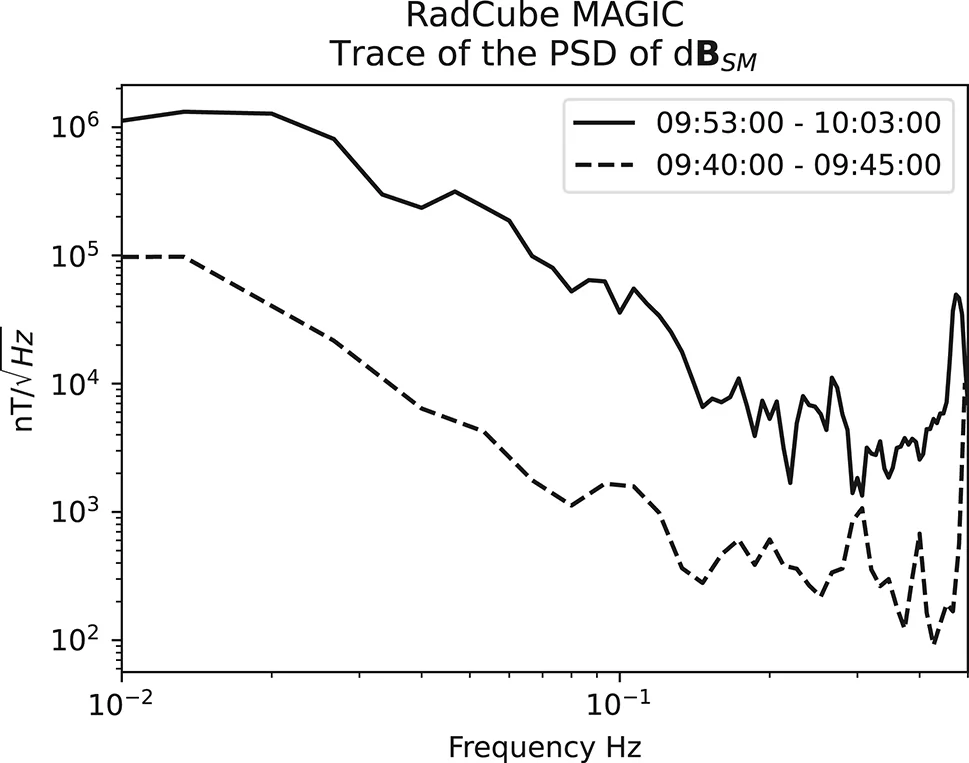
Trace of the power spectral density matrix of d**B** = **B**_RC_ – **B**_IGRF_ for different time intervals during the data shown in Fig. 4. The solid line corresponds to the extended region of magnetic field fluctuations observed after the most intense magnetic field spikes, and the dashed line corresponds to a quiet interval.

To further explore this, Fig. 5 shows the trace of the power spectral density of d**B** = **B**_RC_ – **B**_IGRF_ in the interval 09:53 UT – 10:03 UT (solid line) which corresponds to the extended interval of magnetic field fluctuations in Fig. 4, computed using Welch’s method. For reference, this is compared to a PSD derived from measurements during the quiet conditions observed during the interval 09:40 UT – 09:45 UT (dashed line). This again shows that the FAC fluctuations occupy a broad frequency range (note however that the increase at highest frequencies is due to contamination of the measurement by platform noise as discussed in the Methods section).

We emphasise that Fig. 5 represents a complex spatio-temporal structure and must be interpreted with care. During this time, RadCube moved through a significant range of latitude, and therefore crossed a large region of the ionosphere. Furthermore, during this time the supersubstorm was evolving, and the SuperMAG SML index had almost recovered to pre-supersubstorm levels at the point where the fluctuations were no longer observed. We note that the end of the extended interval of magnetic field fluctuations occurred when RadCube was at low latitudes and so one interpretation is that RadCube exited the region containing significant magnetic field fluctuations. On the other hand, this time also corresponds to the ending of the supersubstorm, and so the end of the fluctuations could be associated with this as well.

**Fig. 6:**
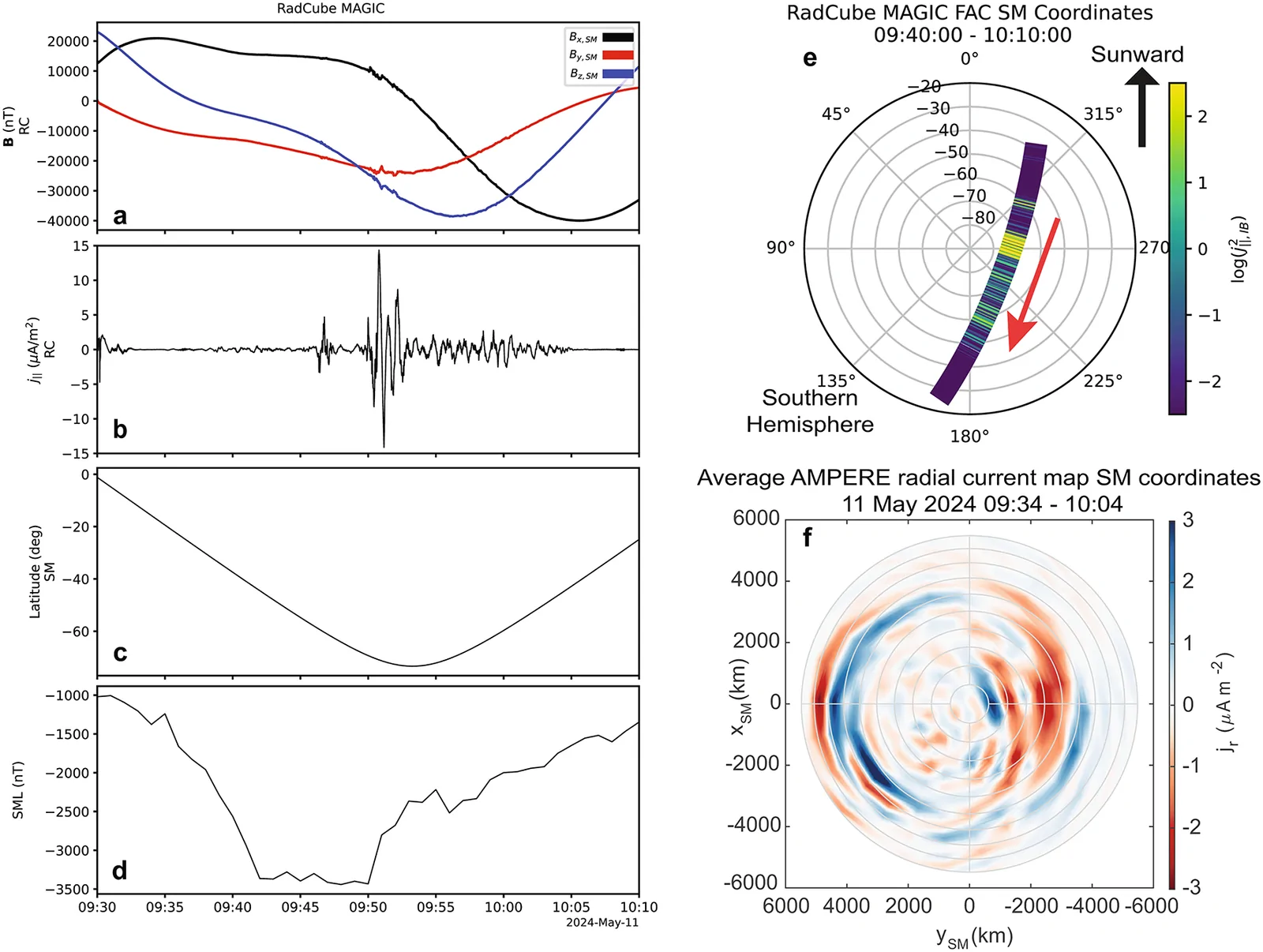
(**a**) RadCube magnetic field measurements in SM coordinate system. (**b**) Derived RadCube field aligned current density. (**c**) RadCube SM latitude. (**d**) SuperMAG SML index. (**e**) RadCube field aligned current density plotted along the RadCube orbit track. The direction of RadCube’s orbital motion is shown by the red arrow. (**f**) Average AMPERE radial current density map in SM coordinates, for the interval 09:34–10:04 UT.

We now use the magnetic field data to estimate the field aligned currents in this event. Figure 6a shows the RadCube magnetic field observations and Fig. 6b shows the associated field aligned current computed from the magnetic field fluctuations (see the Methods section for details about how this single spacecraft approach only applies to scale lengths > 150 km, and time variations > 20 s). FAC spikes reaching a strength of 15 µAm^− 2^ were detected between 09:50 UT – 09:53 UT, corresponding to the largest fluctuations in magnetic field that are visible in Fig. 6a. Figure 6c shows that these intense current spikes were observed at SM latitudes between − 70 ° and − 80 °, at the start of the recovery phase of the supersubstorm based on the SuperMAG SML data shown in Fig. 6d. Figure 6e plots the parameter S = log (j_||_^2^) which is used to identify the location of FACs along the satellite orbit track^40^. Finally, Fig. 6f shows a map of the AMPERE radial field aligned current density, calculated as an average from 09:34–10:04 UT 11 May 2024. Both the location of intense FACs observed by RadCube and the extended region of complex structure FAC signatures are consistent with the AMPERE observations. However, it should be noted that RadCube and AMPERE are complementary in terms of the signatures they capture; this is examined in more detail in the discussion section.

## Discussion

We first consider the magnetic field signatures observed by RadCube. To place the observations in context, we refer to previous studies of extreme events where significant magnetic field fluctuations were also measured. The March 1989 storm is considered to be a 1-in-30 year event^1^, and during this period, observations were made by the EXOS D spacecraft^41^. Intense magnetic field perturbations of the order of 1000 nT were reported, which are comparable to the measurements recorded here.

In addition, in this event the RadCube data also reveals the existence of an extended interval of fluctuating fields, on the nightside and extending to lower latitude. It must be acknowledged that the form of Fig. 5 is reminiscent of a turbulent cascade power law. However, analysis using a single satellite is limited. In these observations Taylor’s hypothesis (i.e. assumptions of time stationarity, as typically used in the analysis of solar wind turbulence) does not apply. This is because the magnetosphere-ionosphere system is evolving quickly during the observations, as evidenced by changes in the SML index. Furthermore, RadCube also sampled a wide range of latitudes/spatial points during this interval. Therefore, fluctuations in time and space are intricately intertwined, and more work on the possible statistical and apparent spectral properties of turbulence in such cases (depending on its source) is required. However, this may also be frustrated by the relatively short time duration of the observations.

The observed fluctuations may arise from at least three sources. Firstly, they could be inherent to the FACs that arise during the extreme conditions here (i.e. due to local instabilities associated with the FACs themselves). Alternatively, they may reflect turbulent driving from the magnetotail or the enhanced magnetospheric convection. Finally, fluctuations may also arise from external turbulent driving from the solar wind and magnetosheath^42^. Despite the fact that the May 2024 events are well observed, the necessary outer magnetosphere measurements on both the day- and night-side are unfortunately not available. The results presented here thus motivate further study of all these aspects of magnetosphere-ionosphere coupling physics during other supersubstorm intervals and may also be a target for simulations.

The RadCube magnetic field data can also be used to infer the structure of FACs. As explained in the Methods section, this applies to structures > 150 km and on time scales > 20 s where assumptions of time stationarity and one-dimensionality are reasonable. It has previously been established that there is no strong relationship between the intensity of a geomagnetic storm and the strength of any supersubstorms that may occur during the storm^16^. The new data from RadCube MAGIC presented here extend this further to considering the FAC strengths that occur during a supersubstorm. The Halloween storms in November 2003 are considered to be a 1-in-10 year event^1^ and observations of FACs by the CHAMP satellite have been reported^43^. CHAMP observed peak FACs of up to 15 µAm^− 2^, which is consistent with the observations presented here. During the September 2017 geomagnetic storm the SWARM satellites observed average FAC densities greater than 3 µAm^− 2^, which are similar in size to the FAC signatures observed by RadCube during a separate period of more minor geomagnetic activity on 30 April 2022^36^. We therefore conclude that the strong FACs observed by RadCube during the May 2024 storm are significant, and comparable to previous observations, but here we document their occurrence during, and therefore their association with, a supersubstorm. Furthermore, the fact that these intense FACs occurred during the recovery phase of the geomagnetic storm provides direct evidence that the phase of a geomagnetic storm is not a simple indicator for the likely strength of FACs that may occur. It also emphasises the importance of predicting supersubstorms during geomagnetic storm intervals.

In terms of monitoring the strengths of FACs, during this event the peak intensities measured by RadCube appear to be somewhat larger than those captured by AMPERE (Fig. 6f). The AMPERE data is based on a fitting procedure applied to engineering-grade magnetic field observations across the Iridium constellation, which because of the methods used to implement the fitting as well as the nature of the underlying magnetic field data means that the minimum resolvable latitudinal scale is ~ 7°^44^. In contrast, and as discussed further in the Methods section, only variations in the RadCube magnetic field measurements on timescales shorter than 100 s can be identified as FACs because of the way in which the data is calibrated. This time scale also corresponds to a latitudinal scale of ~ 7°. Consequently, the RadCube and AMPERE observations provide different, and complementary, views. Whereas AMPERE provides information about the large-scale structure, location and overall pattern of the FACs, RadCube provides detailed local information about the smaller-scale structure along its orbit. This reveals that FACs can be more intense and structured on smaller scales than the AMPERE data may, at first sight, imply.

The importance of these observations for space weather lies in their relevance for understanding the associated GIC signatures that are generated at the surface. As described in the introduction, the May 2024 event was extremely well observed worldwide, and this allows the regional impact of this supersubstorm to be assessed. In particular, at the time of interest, Canada/Alaska was at around midnight MLT. In an analysis of the impacts of the May 2024 storm on the Canadian sector, it was noted that at 09:48 UT on 11 May 2024, strong enhancements were detected in the magnetic field at almost all Canadian geomagnetic observatories, resulting in geoelectric fields exceeding 500 mV/km across the eastern auroral zone^28^. In contrast, analysis of impacts on Sweden showed that the northern European sector on the dayside was not affected by this particular supersubstorm^30^. Overall, we arrive at a picture where the solar wind pressure pulse caused compression of the magnetosphere^38^ and enhancement in magnetospheric convection^37^, driving strong FACs in the auroral region in both hemispheres (observed by RadCube at southerly latitudes). This generated the supersubstorm signatures in geomagnetic indices^35,37^, and resulting ground effects localised in the Canadian sector^28^ on the nightside only.

In summary, the data presented here provide new insight into the nature of FACs during supersubstorms, which represent a significant space weather impact risk through their association with strong GICs on the ground. The observations from RadCube MAGIC reveal both strong localised supersubstorm FACs at high latitude, together with an extended region of fluctuating magnetic fields on the nightside. Previous studies have shown that for events occurring within solar cycle 23, 77% of supersubstorms were associated with solar wind density or pressure enhancements^16^, and so this case study is a relevant example for the most common class of supersubstorm driver.

Finally, we consider the implications of these observations both for future studies into the basic physics of supersubstorms, and for space weather monitoring. With single point observations it is not possible to fully disentangle the spatial and temporal structuring, particularly during a quickly evolving supersubstorm where the satellite was moving rapidly through the FAC region in its orbit. This motivates the need for multipoint LEO measurements such as from TRACERS^45^ that can decouple space and time to improve our understanding of Magnetosphere-Ionosphere coupling. For operational space weather purposes, the results presented here reinforce the conclusion that it is indeed necessary to develop constellation missions in LEO for improved real-time monitoring, as has been proposed elsewhere^46,47^. More specifically, future constellation systems should be capable of resolving and disentangling the magnetic field structure and FACs on temporal and spatial scales associated with supersubstorms since these events are likely to be the source of impactful GICs. Furthermore, the inter-hemispheric variability of supersubstorms remains poorly understood, and such an approach would also allow this question to be investigated in detail. This would require a much more dense measurement network than is currently available, but one which might be achievable with mega-constellations^48^. Because they are designed to act as a telecoms network, the use of mega-constellations to carry hosted payloads such as MAGIC or other miniaturised instrumentation would also directly unlock real-time observation capabilities, which would further improve operational space weather forecasting services.

## Methods

Full details of the MAGIC instrument, which uses Anisotropic Magneto-Resistive (AMR) sensors to detect the magnetic field, and its implementation on the RadCube CubeSat are presented in Ref^36^. The events of May 2024 occurred after the conclusion of the RadCube extended mission in April 2022, and MAGIC was initially non-operational. Based on forecasts of potentially significant space weather impacts, the RadCube project reactivated MAGIC to make magnetic field measurements and test RadCube’s performance after nearly three years in orbit; MAGIC was operational on 11 May 2024 between 07:42 UT − 20:23 UT. This means that observations were captured in the recovery phase of the storm, and successfully demonstrated the ability of RadCube and MAGIC to operate during a severe space weather event. During the observations presented here, RadCube was at an average altitude of 436 km above the surface of the Earth (R_avg_ = 6,807 km in the SM coordinate system) and was in a sun-synchronous polar orbit crossing the SM equatorial plane at a local time of approximately 10:30/22:30 LT.

Magnetic field measurements were made using the MAGIC inboard magnetometer sensor, at a cadence of 1 vector/s with RadCube operating in a magnetically-quiet tumbling mode. The calibration procedure was identical to that described in Sect. 5 of Ref^36^, whereby an attitude-independent calibration procedure was used in combination with reconstruction of the quaternion solution to derive the magnetic field in the Solar Magnetic coordinate system. This method involves applying a low pass filter of 0.01 Hz to the raw data, and so once calibrated only fluctuations above this frequency can be examined for association with field aligned currents. The inboard sensor is mounted on the MAGIC electronics card inside the RadCube, and although the platform was largely magnetically quiet, narrow-band interference is produced at discrete frequencies that are visible in the spectrograms in Fig. 4c, 4e, and 4g. This is also the source of the increase in power at the highest frequencies in Fig. 5. However, this interference does not impact the detection of the magnetic fluctuations and the associated FACs that are the subject of this work.

The calculation of the field aligned current j_||_ is also the same as documented in Sect. 5 of Ref^36^, where the background magnetic field is first calculated by convolving the calibrated data with a 60 s square wave kernel. Calculation of the field aligned current is then based on assuming an infinite 1-d current sheet, and time stationarity. This methodology is most appropriate for large-scale FAC structures, with thicknesses greater than 150 km and durations > 20 s^49^; in performing the calculation the resulting j_||_ is smoothed with a 20 s kernel and so fluctuations at higher frequencies are not directly interpreted in terms of FACs.

In presenting the RadCube observations, we also use the following contextual data:

- OMNI: the OMNI dataset provides the solar wind plasma and magnetic field conditions time shifted to the nose of the Earth’s bow shock^50^. We use the HRO2 product, at 5-minute time resolution in the production of Fig. 1.
- THEMIS (Time History of Events and Macroscale Interactions during Substorms)^51^: The pressure pulse in the solar wind was observed by the THEMIS-C probe in lunar orbit. At this time THEMIS-C was operating in slow survey mode. We show magnetic field data^52^ and solar wind moments from the electrostatic analyser, calculated onboard^53^. During this event, both are measured at a cadence of 4.1 s. The THEMIS-A and THEMIS-E probes in Earth orbit were also both operating in slow survey mode, with only magnetometer data available, at a cadence of 2.7 s and 4 s respectively.
- MMS (Magnetospheric Multiscale)^54^: We show magnetic field^55^ and Fast Plasma Instrument (FPI) ion plasma data^56^ from the MMS1 satellite. MMS1 magnetic field data is available at 16 Hz resolution, with 4.5 s cadence ion moments and ion spectra.
- GOES (Geostationary Operational Environmental Satellite): Magnetic field data from the GOES-16^57^ and GOES-18^58^ satellites are used, at a time resolution of 0.1 s.
- AMPERE (Active Magnetosphere and Planetary Electrodynamics Response Experiment): Magnetic field data was used to compute maps of radial current density^44,59^. Here AMPERE maps are based on an accumulation period of 30 min, as noted in the text, using data from the southern hemisphere.
- SuperMAG^21^: The SuperMAG SML index^20^ quantifies the strength of the electrojet based on the lowest northward component measured across the SuperMAG ground magnetometer network (between 40° and 80° magnetic latitude). SML is similar to the AL index, but because the ground station coverage is more extensive, it more accurately characterises auroral electrojets which descend to lower latitudes during storm intervals^15,20^.
- Sym-H data are used to define the evolution of the geomagnetic superstorm. Whilst both the SYM-H and Dst geomagnetic indices characterise the intensity of the ring current and therefore track the evolution of geomagnetic storms^60^, SYM-H is preferred here as it is available at 1-minute time resolution as opposed to the 1-hour time resolution of Dst.

Data analysis was performed in Python using the PySPEDAS package^61^.

## Data Availability

The RadCube MAGIC magnetic field dataset presented here can be accessed via https://dx.doi.org/10.14469/hpc/14612. AMPERE-NEXT data were obtained from https://ampere.jhuapl.edu/. SuperMAG data were obtained from https://supermag.jhuapl.edu/info. THEMIS, MMS, OMNI, GOES, and SYM-H data were obtained from NASA’s Space Physics Data Facility (SPDF) https://spdf.gsfc.nasa.gov/.

## References

[1] Eastwood, J. P. et al. The economic impact of space weather: where do we stand? *Risk Anal.* **37**, 206–218. 10.1111/risa.12765 (2017). 28230267

[2] Schrijver, C. J. et al. Understanding space weather to shield society: a global road map for 2015–2025 commissioned by COSPAR and ILWS. *Adv. Space Res.* **55**, 2745–2807. 10.1016/j.asr.2015.03.023 (2015).

[3] Hapgood, M. et al. Development of space weather reasonable worst-case scenarios for the UK national risk assessment. *Space Weather*. **19** 10.1029/2020sw002593 (2021).

[4] Cliver, E. W. & Dietrich, W. F. The 1859 space weather event revisited: limits of extreme activity. *J. Space Weather Space Clim.* **3**, 15. 10.1051/swsc/2013053 (2013).

[5] Opgenoorth, H. J. et al. Earth’s geomagnetic environment—progress and gaps in understanding, prediction, and impacts. *Adv. Space Res.* 10.1016/j.asr.2024.05.016 (2024).

[6] Viljanen, A. et al. Recordings of geomagnetically induced currents and a nowcasting service of the Finnish natural gas pipeline system. *Space Weather*. **4** 10.1029/2006sw000234 (2006).

[7] Beggan, C. D. et al. Implementing an operational cloud-based now‐ and forecasting system for space weather ground effects in the UK. *Space Weather*. **23** 10.1029/2025sw004364 (2025).

[8] Oughton, E. J., Skelton, A., Horne, R. B., Thomson, A. W. P. & Gaunt, C. T. Quantifying the daily economic impact of extreme space weather due to failure in electricity transmission infrastructure. *Space Weather*. **15**, 65–83. 10.1002/2016SW001491 (2017).

[9] Eastwood, J. P. et al. Quantifying the economic value of space weather forecasting for power grids: an exploratory study. *Space Weather*. **16**, 2052–2067. 10.1029/2018sw002003 (2018).

[10] Oughton, E. J. et al. A risk assessment framework for the socioeconomic impacts of electricity transmission infrastructure failure due to space weather: an application to the United Kingdom. *Risk Anal.* **39**, 1022–1043. 10.1111/risa.13229 (2019). PMC6936226 30408211

[11] Cowley, S. W. H. Magnetosphere-ionosphere interactions: a tutorial review in. *Magnetospheric Curr. Syst.* 91–106. 10.1029/GM118p0091 (2000).

[12] Kepko, L. et al. Substorm Current Wedge Revisited. *Space Sci. Rev.* **190**, 1–46. 10.1007/s11214-014-0124-9 (2015).

[13] Milan, S. E. et al. Overview of solar wind–magnetosphere–ionosphere–atmosphere coupling and the generation of magnetospheric currents. *Space Sci. Rev.* **206**, 547–573. 10.1007/s11214-017-0333-0 (2017).

[14] Buzulukova, N. & Tsurutani, B. Space weather: from solar origins to risks and hazards evolving in time. *Front. Astronomy Space Sci.* **9** 10.3389/fspas.2022.1017103 (2022).

[15] Tsurutani, B. T., Hajra, R., Echer, E. & Gjerloev, J. W. Extremely intense (SML ≤ -2500 nT) substorms: isolated events that are externally triggered? *Ann. Geophys.* **33**, 519–524. 10.5194/angeo-33-519-2015 (2015).

[16] Hajra, R., Tsurutani, B. T., Echer, E., Gonzalez, W. D. & Gjerloev, J. W. Supersubstorms (SML < – 2500 nT): magnetic storm and solar cycle dependences. *J. Geophys. Res. : Space Phys.* **121**, 7805–7816. 10.1002/2015ja021835 (2016).

[17] Tsurutani, B. T. & Hajra, R. The interplanetary and magnetospheric causes of Geomagnetically Induced Currents (GICs) > 10 A in the Mäntsälä Finland pipeline: 1999 through 2019. *J. Space Weather Space Clim.* **11** 10.1051/swsc/2021001 (2021).

[18] Zong, Q. G., Yue, C. & Fu, S. Y. Shock induced strong substorms and super substorms: preconditions and associated oxygen ion dynamics. *Space Sci. Rev.* **217** 10.1007/s11214-021-00806-x (2021).

[19] Tsurutani, B. T. & Hajra, R. Energetics of shock-triggered supersubstorms (SML < – 2500 nT). *Astrophys. J.* **946** 10.3847/1538-4357/acb143 (2023).

[20] Newell, P. T. & Gjerloev, J. W. Evaluation of SuperMAG auroral electrojet indices as indicators of substorms and auroral power. *J. Geophys. Res. : Space Phys.* **116** 10.1029/2011JA016779 (2011).

[21] Gjerloev, J. W. The SuperMAG data processing technique. *J. Geophys. Res. : Space Phys.* **117** 10.1029/2012JA017683 (2012).

[22] Elvidge, S. & Themens, D. R. The probability of the May 2024 geomagnetic superstorm. *Space Weather*. **23** 10.1029/2024sw004113 (2025).

[23] Oliveira, D. M. et al. Editorial: Impacts of the extreme Gannon geomagnetic storm of May 2024 throughout the magnetosphere-ionosphere-thermosphere system. *Front. Astronomy Space Sci.* **12** 10.3389/fspas.2025.1742847 (2025).

[24] Tulasi Ram, S. et al. Super-intense geomagnetic storm on 10–11 May 2024: possible mechanisms and impacts. *Space Weather*. **22**, e2024SW004126. 10.1029/2024SW004126 (2024). PMC11602687 39620207

[25] Hajra, R., Tsurutani, B. T., Lakhina, G. S., Lu, Q. & Du, A. Interplanetary causes and impacts of the 2024 May superstorm on the geosphere: an overview. *Astrophys. J.* **974** 10.3847/1538-4357/ad7462 (2024).

[26] Ngwira, C. M. et al. Evaluating the geomagnetic response to the May 2024 super storm – observations and interpretations. *Front. Astronomy Space Sci.* **12** 10.3389/fspas.2025.1652705 (2025).

[27] Horne, R. B. et al. The May 2024 geomagnetic storm: UK experience and perspective. *Royal Soc. Open. Sci.* **13** 10.1098/rsos.251943 (2026).

[28] Fiori, R. A. D. et al. A Canadian perspective on the May 2024 space weather event. *J. Space Weather Space Clim.* **15** 10.1051/swsc/2025037 (2025).

[29] Wilkerson, L. A. et al. GIC-related observations during the May 2024 geomagnetic storm in the United States. *Space Weather* **24**, e2025SW004758. 10.1029/2025SW004758 (2026).

[30] Wallner, A. V. L. et al. The geomagnetic storm on 10–12 May 2024 and its effect on the Swedish power grid. *Space Weather*. **24** 10.1029/2025sw004788 (2026).

[31] Lawrence, E. et al. The geomagnetic and geoelectric response to the May 2024 geomagnetic storm in the United Kingdom. *Front. Astronomy Space Sci.* **12** 10.3389/fspas.2025.1550923 (2025).

[32] Fogg, A. R. et al. Sun-to-Mud observations of the May and October storms of 2024: impacts on Ireland’s space weather. *J. Space Weather Space Clim.* **16** 10.1051/swsc/2025044 (2026).

[33] Wang, J. et al. The new geomagnetic monitoring network in China: insights from the 2024 mother’s day superstorm. *Space Weather*. **24** 10.1029/2025sw004610 (2026).

[34] Clilverd, M. A. et al. Geomagnetically induced currents, transformer harmonics, and reactive power impacts of the Gannon storm in May 2024. *Space Weather*. **23** 10.1029/2024sw004235 (2025).

[35] Hajra, R., Tsurutani, B. T., Lu, Q., Du, A. & Lakhina, G. S. Supersubstorms during the May 2024 superstorm. *J. Space Weather Space Clim.* **15** 10.1051/swsc/2025047 (2025).

[36] Eastwood, J. P. et al. In flight performance of the MAGIC magnetoresistive magnetometer on the RadCube cubesat. *Space Sci. Rev.* **221** 10.1007/s11214-025-01170-w (2025). PMC12078372 40386150

[37] Zou, Y. et al. Are supersubstorms substorms? Extreme nightside auroral electrojet activities during the May 2024 geomagnetic storm. *J. Geophys. Res. : Space Phys.* **130** 10.1029/2024ja033303 (2025).

[38] Liu, T. Z. et al. Near-Earth reconnection contributing to recovery phase of geomagnetic storm. *Geophys. Res. Lett.* **51** 10.1029/2024gl112730 (2024).

[39] Farris, M. H. & Russell, C. T. Determining the standoff distance of the bow shock: Mach number dependence and use of models. *J. Geophys. Res.* **99**, 17681–17689. 10.1029/94JA01020 (1994).

[40] Heilig, B. & Lühr, H. New plasmapause model derived from CHAMP field-aligned current signatures. *Ann. Geophys.* **31**, 529–539. 10.5194/angeo-31-529-2013 (2013).

[41] Fujii, R. et al. Field-aligned current signatures during the March 13–14, 1989, Great Magnetic Storm. *J. Geophys. Res. : Space Phys.* **97**, 10703–10715. 10.1029/92JA00171 (1992).

[42] Telloni, D. et al. Does turbulence along the coronal current sheet drive ion cyclotron waves? *Astrophys. J.* **944** 10.3847/1538-4357/acb693 (2023).

[43] Wang, H. et al. Field-aligned currents observed by CHAMP during the intense 2003 geomagnetic storm events. *Ann. Geophys.* **24**, 311–324. 10.5194/angeo-24-311-2006 (2006).

[44] Waters, C. L. et al. Science data products for AMPERE in *Ionospheric Multi-Spacecraft Analysis Tools: Approaches for Deriving Ionospheric Parameters*, 141–165 (2020). 10.1007/978-3-030-26732-2_7

[45] Halekas, J. S. et al. Electron dispersion at the electron edge of the Earth’s magnetospheric cusp. *Geophys. Res. Lett.* **53**, e2026GL122699. 10.1029/2026GL122699 (2026).

[46] Morley, S. K. Challenges and opportunities in magnetospheric space weather prediction. *Space Weather*. **18** 10.1029/2018sw002108 (2020).

[47] Kraft, S., Lupi, A. & Luntama, J. P. ESA’s distributed space weather sensor system (D3S) utilizing hosted payloads for operational space weather monitoring. *Acta Astronaut.* **156**, 157–161. 10.1016/j.actaastro.2018.01.020 (2019).

[48] Archer, M. et al. (ed, O.) First detection of field-aligned currents using engineering magnetometers from the OneWeb mega-constellation. *Space Weather* **23** e2025SW004573 10.1029/2025SW004573 (2025).

[49] McGranaghan, R. M., Mannucci, A. J. & Forsyth, C. A comprehensive analysis of multiscale field-aligned currents: characteristics, controlling parameters, and relationships. *J. Geophys. Res. : Space Phys.* **122** 10.1002/2017ja024742 (2017).

[50] Papitashvili, N. E., King, J. H. & OMNI Combined solar wind plasma moments and interplanetary magnetic field (IMF) time-shifted to the nose of the Earth’s bow shock, plus geomagnetic indices, 5 min Data. (2020). 10.48322/hkaw-ff03

[51] Angelopoulos, V. The THEMIS mission. *Space Sci. Rev.* **141**, 5–34. 10.1007/s11214-008-9336-1 (2008).

[52] Auster, H. U. et al. The THEMIS fluxgate magnetometer. *Space Sci. Rev.* **141**, 235–264. 10.1007/s11214-008-9365-9 (2008).

[53] McFadden, J. P. et al. The THEMIS ESA plasma instrument and in-flight calibration. *Space Sci. Rev.* **141**, 277–302. 10.1007/s11214-008-9440-2 (2008).

[54] Burch, J. L., Moore, T. E., Torbert, R. B. & Giles, B. L. Magnetospheric Multiscale overview and science objectives. *Space Sci. Rev.* **199**, 5–21. 10.1007/s11214-015-0164-9 (2016).

[55] Russell, C. T. et al. The Magnetospheric Multiscale magnetometers. *Space Sci. Rev.* **199**, 189–256. 10.1007/s11214-014-0057-3 (2016).

[56] Pollock, C. et al. Fast plasma investigation for Magnetospheric Multiscale. *Space Sci. Rev.* **199**, 331–406. 10.1007/s11214-016-0245-4 (2016).

[57] Loto’aniu, T. M. et al. The GOES-16 spacecraft science magnetometer. *Space Sci. Rev.* **215** 10.1007/s11214-019-0600-3 (2019).

[58] Loto’aniu, P. T. M. et al. Initial on-orbit results from the GOES-18 spacecraft science magnetometer. *Space Sci. Rev.* **219** 10.1007/s11214-023-01032-3 (2023).

[59] Anderson, B. J. et al. Iridium communications satellite constellation data for study of Earth’s magnetic field. *Geochem. Geophys. Geosyst.* **22**, e2020GC009515. 10.1029/2020GC009515 (2021).

[60] Wanliss, J. A. & Showalter, K. M. High-resolution global storm index: Dst versus SYM-H. *J. Geophys. Res. : Space Phys.* **111** 10.1029/2005JA011034 (2006).

[61] Grimes, E. W. et al. The space physics environment data analysis system in python. *Front. Astronomy Space Sci.* **9** 10.3389/fspas.2022.1020815 (2022). PMC6380193 30880847

